# Clinical Characteristics and Prognostic Analysis of Extramedullary Disease in Multiple Myeloma: A 15-Year Retrospective Cohort Study

**DOI:** 10.3390/cancers18152484

**Published:** 2026-08-03

**Authors:** Jingliang Zhao, Qirui Bai, Qile Qiu, Jiaying Song, Siyu Kong, Kun Zhu, Yifan Zhang, Shengtao Li, Yanping Ma, Lin Zhang, Xiaoqi Qin

**Affiliations:** Department of Hematology, The Second Hospital of Shanxi Medical University, 382 Wuyi Road, Taiyuan 030000, China; 18834856257@163.com (J.Z.);

**Keywords:** multiple myeloma, extramedullary disease, bone-related extramedullary disease, soft-tissue extramedullary disease, prognosis

## Abstract

Background: Extramedullary disease (EMD) in multiple myeloma (MM) is associated with poor prognosis, yet the clinical distinctions between bone-related EMD (bEMD) and soft tissue-associated EMD (sEMD) remain incompletely characterized. This study aimed to compare the clinical features and outcomes of bEMD versus sEMD and to develop a simple pre-treatment risk stratification tool using baseline clinical parameters. Methods: We retrospectively analyzed 118 patients with MM and EMD treated at a single center from 2011 to 2025, including 72 bEMD and 46 sEMD cases. Baseline characteristics, laboratory parameters, cytogenetic abnormalities, and survival outcomes were compared. Results: Patients with sEMD had significantly higher serum β2-microglobulin (β2-MG) levels (6.4 mg/L vs. 4.5 mg/L, *p* = 0.007) and a higher proportion of relapsed/refractory disease (41.3% vs. 15.3%, *p* = 0.002) compared to bEMD. Median progression-free survival (PFS) and overall survival (OS) were markedly shorter in patients with sEMD than in those with bEMD (PFS: 12.0 vs. 29.0 months, *p* < 0.001; OS: 25.0 vs. 67.0 months, *p* = 0.009). Multivariate analysis identified thrombocytopenia (PLT < 100 × 109/L), elevated β2-MG, multisite extramedullary involvement, and TP53 deletion as independent adverse prognostic factors. A risk scoring system incorporating β2-MG (0–2 points), thrombocytopenia (1 point), and multisite involvement (1 point) stratified patients into low-risk (0–2 points) and high-risk (3–4 points) groups with significantly different PFS (27.0 vs. 10.0 months, *p* < 0.001) and OS (54.0 vs. 22.0 months, *p* = 0.008). Conclusions: sEMD represents a more aggressive subtype of MM with inferior outcomes. The proposed risk score, based on routinely available clinical parameters, effectively identifies high-risk patients at initial diagnosis and may guide individualized treatment strategies.

## Simple Summary

Extramedullary disease is a severe form of multiple myeloma in which myeloma cells grow outside the bone marrow. However, not all types of extramedullary disease behave in the same way. In this study, we reviewed 118 patients with multiple myeloma and extramedullary disease treated at our center over a 15-year period. We found that patients with soft tissue-associated extramedullary disease had more aggressive clinical features and shorter survival than those with bone-related extramedullary disease. We also identified several routinely available clinical factors, including elevated β2-microglobulin, thrombocytopenia, and involvement of multiple extramedullary sites, that were associated with poor outcomes. Based on these factors, we developed a simple risk scoring system that may help clinicians identify high-risk patients at diagnosis and support more individualized treatment decisions.

## 1. Introduction

Multiple myeloma (MM) is a plasma cell neoplasm driven by the clonal expansion of malignant plasma cells and typically presents with infections, bone pain, anemia, renal impairment, and hypercalcemia [1]. In recent years, patient outcomes in MM have improved substantially, owing to advances in immunotherapy, autologous hematopoietic stem cell transplantation, and the continued development of novel targeted agents. Nevertheless, a subset of patients continues to experience a poor prognosis, characterized by rapid disease progression and refractoriness to standard therapies. Among the key adverse prognostic factors in this population, the emergence of extramedullary disease (EMD) represents a major driver of poor outcomes [2]. Extramedullary disease (EMD) in multiple myeloma is broadly categorized into soft tissue-associated EMD (sEMD) and bone-related EMD (bEMD), based on the anatomic relationship of the lesion to osseous structures. bEMD typically arises from direct cortical disruption and is confined to adjacent soft tissues, whereas sEMD can disseminate hematogenously to distant sites, including the lungs, lymph nodes, thyroid, kidneys, peritoneum, pleura, and central nervous system [3]. Emerging evidence indicates that patients with sEMD have significantly poorer outcomes than those with bEMD [4]. Despite increasing recognition of EMD as a high-risk manifestation of MM, the biological mechanisms underlying extramedullary dissemination and the clinical heterogeneity among different EMD subtypes remain incompletely understood. Recent studies have highlighted the contributions of altered tumor–microenvironment interactions, loss of bone marrow niche dependence, and high-risk molecular features to extramedullary progression. However, reliable pretreatment tools specifically designed for risk stratification of patients with EMD remain limited [5,6]. In this study, we performed a retrospective analysis of 118 patients with multiple myeloma and extramedullary involvement to delineate the clinical features and prognostic relevance of bEMD versus sEMD. Our primary objective was to evaluate whether routinely available laboratory parameters at initial diagnosis could be leveraged to construct a prognostic risk stratification tool—one that remains informative regardless of subsequent treatment intensity or regimen complexity. Such a tool may facilitate early identification of high-risk individuals and support the development of tailored therapeutic strategies.

## 2. Patients and Methods

### 2.1. Patients

This retrospective cohort study included 118 patients diagnosed with MM and EMD at the Second Hospital of Shanxi Medical University between August 2011 and August 2025. EMD can be classified into two types based on the site of infiltration: sEMD, which refers to plasma cell infiltration in soft tissues or organs distant from the BM through hematogenous dissemination, and bEMD, which is extramedullary plasmacytoma that extends directly through the destruction of the bone cortex and is adjacent to the bone tissue. Patients were categorized into bEMD (*n* = 72) and sEMD (*n* = 46) groups based on anatomical characteristics of extramedullary lesions. Among the 118 patients, 10 had concurrent bEMD and sEMD manifestations and were classified into the sEMD group according to the prespecified hierarchical rule.

Inclusion criteria were: (1) confirmed diagnosis of MM per the 2014 International Myeloma Working Group (IMWG) criteria [7]; (2) imaging evidence (via MRI or PET-CT) of extramedullary involvement adjacent to bone, within soft tissues, or in solid organs, confirmed by histopathological identification of plasmacytoma at the lesion site or radiographic findings; and (3) availability of complete follow-up data. Patients with both bEMD and sEMD manifestations were assigned to the sEMD group. Individuals with plasma cell leukemia or solitary bone plasmacytoma were excluded from the study.

### 2.2. Methods

Baseline demographic, clinical, and laboratory characteristics were compared between the bEMD and sEMD groups at initial diagnosis. These included age, sex, and β2-MG levels, as well as the frequencies of anemia, thrombocytopenia, elevated lactate dehydrogenase (LDH), renal insufficiency, multisite extramedullary involvement, high bone marrow plasma cell (BMPC) infiltration, newly diagnosed multiple myeloma (NDMM) with EMD, relapsed and/or refractory multiple myeloma (RRMM) with EMD, M-protein type, light chain type, Durie–Salmon (DS) stage, International Staging System (ISS) [8], and relevant cytogenetic abnormalities.

The following predefined cutoffs were applied: thrombocytopenia (PLT < 100 × 10^9^/L), anemia (Hb < 100 g/L), renal insufficiency (serum creatinine ≥ 177 umol/L), LDH ≥ 250 U/L, multisite extramedullary involvement (EMD involving ≥ 2 distinct anatomical sites), and high BMPC infiltration (≥50%).

All laboratory and cytogenetic data were obtained retrospectively from electronic medical records at the Second Hospital of Shanxi Medical University. Routine clinical parameters—including complete blood counts, serum biochemistry, β2-MG, and LDH—were measured in the institutional clinical laboratory following standard operating procedures. Cytogenetic alterations, including IGH rearrangements, TP53 deletion, RB1 deletion, and 1q21 gain/amplification, were assessed by fluorescence in situ hybridization (FISH) on purified plasma cell samples, as part of routine diagnostic workup in the hospital’s pathology/hematology laboratory.

### 2.3. Follow-Up

Follow-up was continued until death or 1 December 2025, whichever occurred first. Follow-up assessments were conducted primarily through outpatient clinic visits, supplemented by telephone interviews and review of inpatient medical records. Progression-free survival (PFS) was defined as the time from initial diagnosis to disease progression, relapse, death from any cause, or the last follow-up, whichever occurred first. Overall survival (OS) was measured from the date of diagnosis to the date of death from any cause or censoring at the final follow-up.

### 2.4. Statistical Analysis

Data analysis was performed using SPSS version 27.0 (IBM Corp., Armonk, NY, USA) and R software version 4.2.1 (R Foundation for Statistical Computing, Vienna, Austria). PFS and OS were estimated using the Kaplan–Meier method, with comparisons performed using the log-rank test. Continuous variables were expressed as median (interquartile range) and compared using the Mann–Whitney U test. Categorical variables were analyzed using the χ^2^ test or Fisher’s exact test, as appropriate. Prognostic factors were assessed via univariate and multivariate Cox proportional hazards regression models. Variables with *p* < 0.05 in univariate analysis were entered into multivariate models. Hazard ratios (HR) with 95% confidence intervals (CI) were calculated. Model performance was evaluated using concordance index (C-index), likelihood ratio testing, Akaike information criterion (AIC), and time-dependent receiver operating characteristic (ROC) analysis. The proportional hazards assumption was assessed using Schoenfeld residuals with the cox.zph function. All statistical tests were two-sided, and *p* < 0.05 was considered statistically significant. To assess model stability, internal validation was performed using bootstrap resampling with 1000 iterations.

## 3. Results

### 3.1. Clinical Characteristics

Among the 118 patients with multiple myeloma and EMD, 72 (61.0%) were classified as bEMD and 46 (39.0%) as sEMD. The median age at diagnosis was comparable between the bEMD and sEMD groups (59.5 years [IQR 52–66] vs. 61.0 years [IQR 55–69]; z = −1.403, *p* = 0.161). Sex distribution differed significantly: the bEMD group was predominantly male (63.9%), whereas the sEMD group was predominantly female (60.9%; χ^2^ = 6.932, *p* = 0.008). β2-MG levels were significantly higher in the sEMD group (median 6.4 mg/L [IQR 4.0–10.0]) than in the bEMD group (median 4.5 mg/L [IQR 2.8–7.4]; *p* = 0.007). Furthermore, newly diagnosed MM (NDMM) with EMD was more frequent in the bEMD group, while relapsed/refractory MM (RRMM) with EMD was more common in the sEMD group (χ^2^ = 10.028, *p* = 0.002). No statistically significant differences were observed between the two groups with respect to anemia, thrombocytopenia, elevated LDH, renal insufficiency, BMPC ≥ 50%, M-protein type, light chain type, DS stage, ISS staging, or the presence of multisite extramedullary involvement (all *p* > 0.05). However, the sEMD group exhibited a higher trend toward thrombocytopenia (23.9% vs. 11.1%, *p* = 0.065) and elevated LDH (34.8% vs. 19.4%, *p* = 0.062). Cytogenetic profiles, including IGH rearrangements, TP53 deletion, and RB1 deletion, did not differ significantly between the two groups (Table 1). Although 1q21 gain/amplification was more frequent in the bEMD group (49.0%, 24/49) than in the sEMD group (30.0%, 9/30), this difference did not reach statistical significance (χ^2^ = 2.756, *p* = 0.097). Immunohistochemical analysis revealed a higher Ki-67 proliferation index in sEMD tumors, though not significantly (*p* = 0.734). CD56 negativity was more frequently observed in the sEMD group (72.2%, 26/36) than in the bEMD group (51.9%, 28/54), a difference that approached but did not achieve statistical significance (χ^2^ = 3.735, *p* = 0.053). Regarding treatment patterns, no substantial treatment-related imbalance was observed between the sEMD and bEMD groups.

### 3.2. Survival Analysis

**Figure 1:**
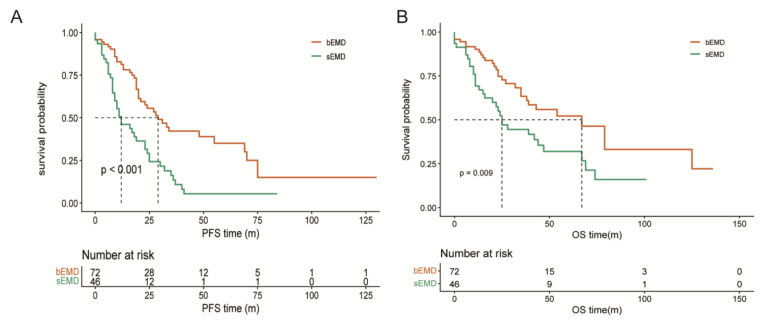
Survival outcomes of bEMD versus sEMD patients. (**A**) Kaplan–Meier curves showing progression-free survival (PFS) in bEMD (*n* = 72) vs. sEMD (*n* = 46) groups. (**B**) Overall survival (OS) in bEMD vs. sEMD groups. *p*-values were calculated using the log-rank test.

Survival analyses were performed to systematically evaluate the prognostic impact of clinical and biological factors in 118 patients with MM and EMD. Patients in the bEMD group exhibited significantly longer median PFS than those in the sEMD group (29.0 months [95% CI: 18.4–39.6] vs. 12.0 months [95% CI: 4.4–19.6]; *p* < 0.001; Figure 1A). Median OS was similarly prolonged in the bEMD group (67.0 months [95% CI: 40.1–93.9] vs. 25.0 months [95% CI: 18.0–32.0]; *p* = 0.009; Figure 1B). Kaplan–Meier analysis integrating EMD subtype with disease status further confirmed that patients with sEMD had inferior outcomes compared with those with bEMD (Figure S1).

**Figure 2:**
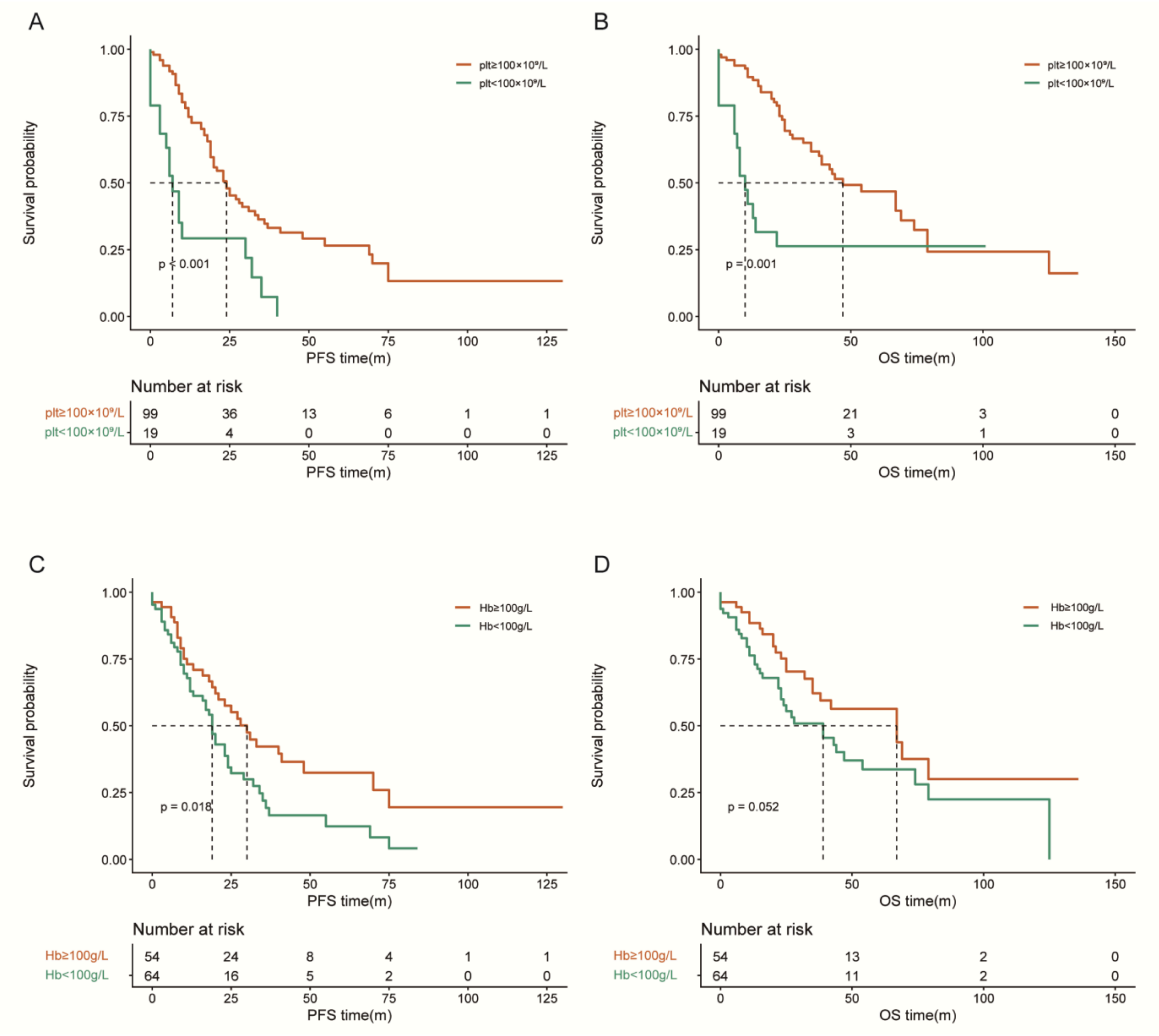
Impact of thrombocytopenia and anemia on survival. (**A**) PFS in patients with thrombocytopenia (PLT < 100 × 10^9^/L) vs. normal platelet count. (**B**) OS in patients with thrombocytopenia vs. normal platelet count. (**C**) PFS in patients with anemia (Hb < 100 g/L) vs. normal hemoglobin. (**D**) OS in patients with anemia vs. normal hemoglobin.

We next assessed the prognostic relevance of hematologic parameters, tumor burden, and cytogenetic abnormalities. Among hematologic variables, thrombocytopenia (PLT < 100 × 10^9^/L; *n* = 19) was associated with markedly inferior outcomes. Median PFS was 7.0 months (95% CI: 3.8–10.2) in thrombocytopenic patients vs. 24.0 months (95% CI: 17.7–30.3) in those with normal platelet counts (*p* < 0.001; Figure 2A), and median OS was 10.0 months (95% CI: 5.7–14.3) vs. 47.0 months (95% CI: 36.3–57.7), respectively (*p* = 0.001; Figure 2B). Disease progression occurred in 89.5% (17/19) of patients in the thrombocytopenic group. Anemia (Hb < 100 g/L; *n* = 64) was also associated with shorter PFS (19.0 months [95% CI: 16.0–22.0] vs. 30.0 months [95% CI: 21.5–38.5]; *p* = 0.018; Figure 2C), with a trend toward reduced OS that did not reach statistical significance (39.0 months [95% CI: 19.1–58.9] vs. 67.0 months [95% CI: 28.3–105.7]; *p* = 0.052; Figure 2D).

**Figure 3:**
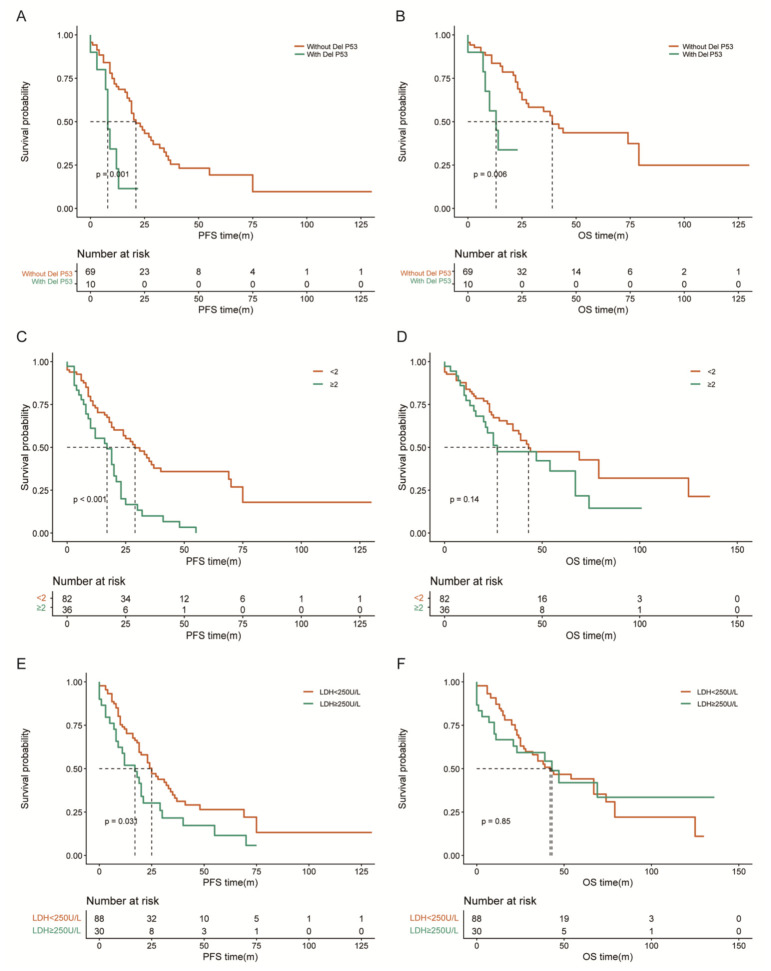
Prognostic impact of TP53 deletion, multifocal involvement, and elevated LDH. (**A**) PFS by TP53 deletion status. (**B**) OS by TP53 deletion status. (**C**) PFS by multifocal extramedullary involvement. (**D**) OS by multifocal extramedullary involvement. (**E**) PFS by LDH level. (**F**) OS by LDH level.

With respect to tumor burden and biological aggressiveness, TP53 deletion (*n* = 10) conferred a particularly poor prognosis. Patients harboring TP53 deletion exhibited significantly shorter PFS (8.0 months [95% CI: 6.1–9.9] vs. 21.0 months [95% CI: 16.3–25.7]; *p* = 0.001) and OS (13.0 months [95% CI: 4.3–21.7] vs. 39.0 months [95% CI: 28.8–49.3]; *p* = 0.006) (Figure 3A,B). PFS and OS events occurred in 80% and 60% of TP53-deleted patients, respectively. Multisite extramedullary involvement (≥2 distinct sites; *n* = 36) was associated with significantly shorter PFS (17.0 months [95% CI: 9.4–24.6] vs. 29.0 months [95% CI: 19.5–38.5]; *p* < 0.001; Figure 3C), though the difference in OS did not achieve statistical significance (27.0 months [95% CI: 0–54.2] vs. 43.0 months [95% CI: 16.8–69.2]; *p* = 0.137; Figure 3D). Elevated LDH (*n* = 30) was likewise associated with shorter PFS (17.0 months [95% CI: 7.8–26.2] vs. 25.0 months [95% CI: 18.5–31.5]; *p* = 0.031; Figure 3E), but no significant difference in OS was observed (43.0 vs. 42.0 months; *p* = 0.854; Figure 3F).

### 3.3. Univariate and Multivariate Analysis of Factors Affecting PFS in MM Patients with EMD

Univariate and multivariate Cox proportional hazards regression analyses were performed to identify factors associated with PFS in patients with MM and EMD. Candidate variables included age groups, thrombocytopenia, multisite extramedullary involvement, EMD subtype (bEMD vs. sEMD), β2-MG, TP53 deletion, BMPC infiltration ≥ 50%, anemia, elevated LDH, induction regimens and transplantation (Table S1). Given that TP53 deletion data were available in only 79 of 118 patients (66.9%), two separate multivariate models were constructed to derive a robust prognostic framework based on the maximum available sample size. In the first model, which included all 118 patients and excluded TP53 deletion, multisite extramedullary involvement (HR = 2.23, 95% CI: 1.34–3.71, *p* = 0.002), thrombocytopenia (HR = 2.57, 95% CI: 1.37–4.82, *p* = 0.003), and serum β2-MG (per 1 mg/L increase, HR = 1.08, 95% CI: 1.05–1.11, *p* < 0.001) emerged as independent predictors of shorter PFS (Table 2). Elevated LDH did not retain statistical significance in this model (HR = 1.51, 95% CI: 0.90–2.55, *p* = 0.12). In the subgroup of 79 patients with complete TP53 deletion data, multivariate analysis identified TP53 deletion (HR = 2.88, 95% CI: 1.22–6.79, *p* = 0.016), thrombocytopenia (HR = 3.54, 95% CI: 1.71–7.33, *p* < 0.001), elevated LDH (HR = 2.53, 95% CI: 1.38–4.63, *p* = 0.003), and serum β2-MG (per 1 mg/L increase, HR = 1.09, 95% CI: 1.05–1.12, *p* < 0.001) as independent adverse prognostic factors for PFS.

### 3.4. Univariate and Multivariate Analysis of Factors Affecting OS in MM Patients with EMD

Analogous univariable and multivariable Cox regression analyses were performed for OS (Table S2). In the full cohort (*n* = 118, excluding TP53 deletion), thrombocytopenia (HR = 2.68, 95% CI: 1.45–4.94, *p* = 0.002) and serum β2-MG (per 1 mg/L increase, HR = 1.08, 95% CI: 1.05–1.12, *p* < 0.001) were independently associated with inferior OS (Table 3). In the TP53 evaluable subgroup (*n* = 79), TP53 deletion (HR = 3.42, 95% CI: 1.29–9.09, *p* = 0.014), thrombocytopenia (HR = 2.76, 95% CI: 1.26–6.03, *p* = 0.011), and serum β2-MG (per 1 mg/L increase, HR = 1.10, 95% CI: 1.06–1.14, *p* < 0.001) each retained independent prognostic significance for OS.

### 3.5. Development and Exploratory Analysis of a Prognostic Risk Scoring System

Based on the independent prognostic factors identified for PFS, a simplified risk scoring system was constructed. The model was developed as follows: variables retaining independent prognostic significance for PFS in multivariate analysis—namely serum β2-MG, thrombocytopenia, and multisite extramedullary involvement—were incorporated into the scoring algorithm. Multisite extramedullary involvement and thrombocytopenia were each assigned a score of 1 point. Serum β2-MG was categorized and scored as 0 (<3.5 mg/L), 1 (3.5–5.5 mg/L), or 2 points (>5.5 mg/L). Based on the total score, the 118 patients were stratified into a low-risk group (0–2 points; *n* = 95) and a high-risk group (3–4 points; *n* = 23) (Table 4).

**Figure 4:**
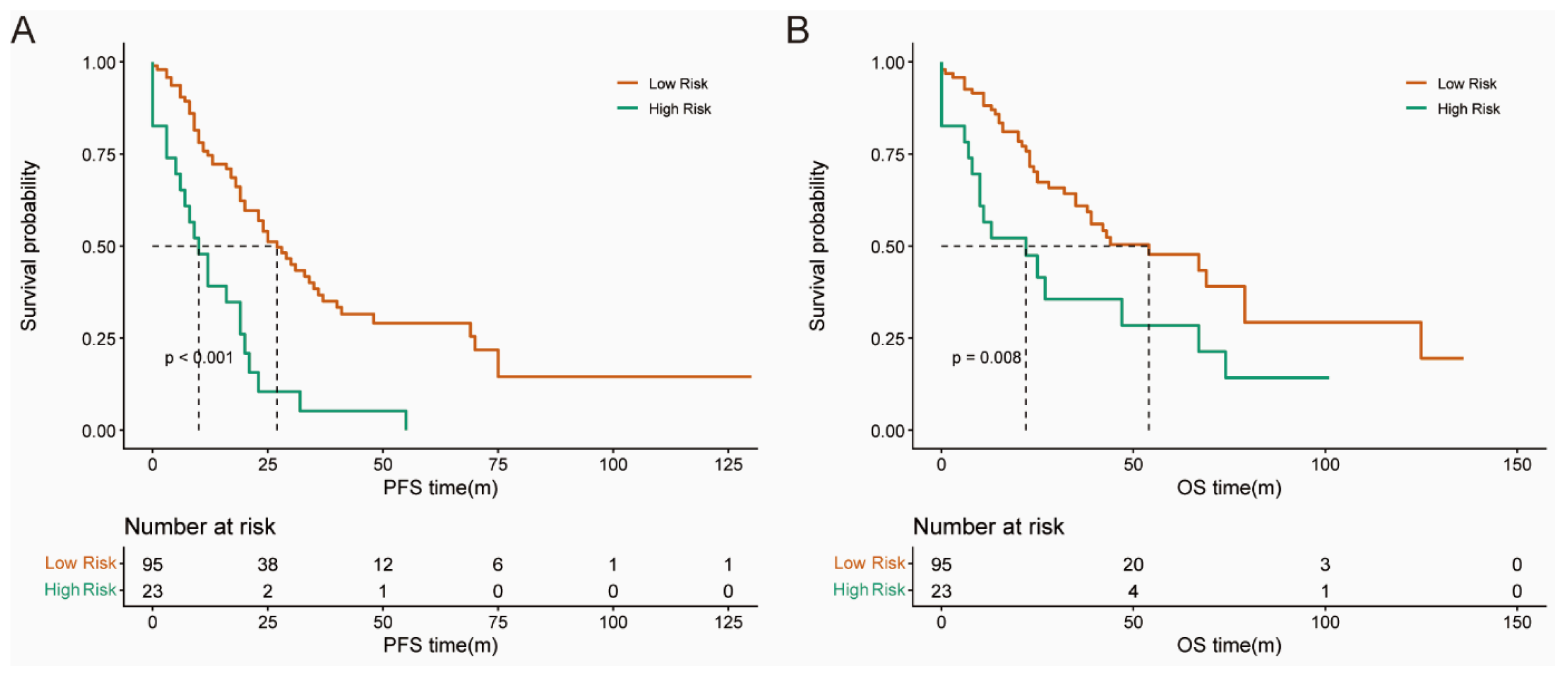
Risk stratification using the prognostic scoring system. (**A**) PFS in low-risk (0–2 points) (*n* = 95) vs. high-risk (3–4 points) (*n* = 23) groups. (**B**) OS in low-risk vs. high-risk groups.

This scoring system demonstrated significant discriminatory capacity. Patients in the high-risk group exhibited markedly shorter median PFS compared to those in the low-risk group (10.0 months vs. 27.0 months; *p* < 0.001; Figure 4A). Although the independent prognostic factors for OS differed somewhat from those for PFS, the PFS-based scoring system nevertheless effectively distinguished OS outcomes: median OS was 22.0 months (95% CI: 2.8–41.2) in the high-risk group versus 54.0 months (95% CI: 26.4–81.6) in the low-risk group (*p* = 0.008; Figure 4B).

To preliminarily assess the stability of model parameters, internal validation was performed using bootstrap resampling (1000 iterations). For the PFS model, both multisite extramedullary involvement (B = 0.833; 95% CI: 0.403–1.277) and thrombocytopenia (B = 0.852; 95% CI: 0.296–1.658) demonstrated robust performance across bootstrap samples. In contrast, the β2-MG score showed relatively limited stability in its independent contribution (B = 0.126; 95% CI: –0.147 to 0.421) (Table 5). For the OS model, conventional multivariate analysis identified only thrombocytopenia as an independent prognostic factor (HR = 2.269; *p* = 0.012); bootstrap validation suggested that baseline characteristics alone provide limited independent predictive capacity for OS (Table 6). Schoenfeld residual analysis showed that although thrombocytopenia alone suggested a potential deviation from the proportional hazards assumption, the global model test did not demonstrate a significant violation of this assumption (Table S3).

To determine whether the scoring system provided additional prognostic information beyond ISS staging, model performance was evaluated for both PFS and OS outcomes. For PFS, the C-index of the scoring system was 0.602 (SE, 0.026), compared with 0.531 (SE, 0.032) for the ISS model. Likelihood ratio testing showed that the risk score model significantly improved model performance compared with ISS staging (χ^2^ = 14.192, *p* < 0.001). Furthermore, the risk score model showed a lower AIC value than the ISS model (625.08 vs. 641.27). Time-dependent ROC analysis further supported the superior predictive ability of the risk score model, with higher AUC values at both 3 years (0.622 vs. 0.583) and 5 years (0.640 vs. 0.528) compared with ISS staging. However, no clear advantage over ISS staging was observed for OS prediction (Table S3).

## 4. Discussion

Extramedullary disease (EMD) represents a high-risk clinical feature in multiple myeloma (MM) and is associated with poor prognosis. It has emerged as a prominent area of investigation in hematologic oncology [4]. Based on anatomical relationship to adjacent bone structures, EMD is categorized into bone-related EMD (bEMD) and soft tissue-associated EMD (sEMD), two entities that differ substantially in pathogenesis, clinical presentation, and prognostic outcomes [9]. Recent real-world evidence has further demonstrated substantial heterogeneity in clinical outcomes according to the timing and pattern of extramedullary involvement, underscoring the importance of more refined EMD classification and risk assessment [10]. These differences may reflect distinct biological processes involved in plasma cell dissemination and adaptation outside the bone marrow niche. Recent genomic and microenvironmental studies have suggested that EMD development is associated with complex interactions between malignant plasma cells, adhesion pathways, immune components, and stromal environments, highlighting the need for integrated approaches to improve risk assessment [11,12]. In an analysis of the SEER database involving 155 patients with MM and EMD, 99 (63.9%) presented with bEMD and 56 (36.1%) with sEMD [13]. Similarly, in the present cohort of 118 EMD patients, 72 (61.0%) had bEMD and 46 (39.0%) had sEMD. Guliyev et al. [14] described a series of 132 EMD cases, of which 93 (70.5%) were classified as bEMD and 39 (29.5%) as sEMD. In their study, EMD was identified at initial diagnosis in 98 patients (74.2%) and at relapse in 34 patients (25.8%). In our cohort, EMD was present at diagnosis in 88 patients (74.6%) and at disease recurrence in 30 patients (25.4%)—findings with proportions nearly identical to those reported by Guliyev et al.

In the present cohort, male patients accounted for 54.2% (64/118) of individuals with MM and concomitant EMD, a modest predominance consistent with prior retrospective studies reporting higher incidence and mortality rates among men in this population [15]. However, sex-specific differences between bEMD and sEMD subgroups remain poorly characterized. Notably, in our cohort, the proportion of male patients was significantly higher in the bEMD group (63.9%) than in the sEMD group (39.1%), whereas the sEMD group was predominantly female (60.9%; *p* = 0.008). The biological mechanisms underlying this disparity are not yet fully elucidated. It has been suggested that 17β-estradiol may potentiate the immunosuppressive function of myeloid-derived suppressor cells (MDSCs), thereby promoting MM progression [16]. This raises the possibility that higher estrogen levels in women could contribute to a more immunosuppressive microenvironment within soft tissues, potentially facilitating tumor cell colonization at extramedullary sites. Additionally, MAST4, an estrogen-responsive gene, has been implicated in the regulation of MM-associated bone disease, and its differential expression may influence the capacity of malignant plasma cells to disseminate to extramedullary compartments [17]. A recent study leveraging exosomal non-coding RNA expression profiles has demonstrated that the female non-hyperdiploid subtype directly drives disease progression and therapeutic resistance through upregulation of the oncogenes MYC and JUN. Concurrently, female-specific dysregulation of NFAT5 mRNA and downregulation of cancer-associated pathways (including LAMC3, GLI2, and TCF7) have been linked to altered cell adhesion, tumor dissemination, and resistance to apoptosis in MM cells [18]. At the same time, the pathogenesis of sEMD is thought to involve the acquisition by plasma cells of survival capacity independent of the bone marrow niche, as well as activation of epithelial–mesenchymal transition (EMT) [19]. Whether these processes are subject to sex-specific regulation remains an open question and warrants further investigation.

In the present study, median serum β2-MG levels were significantly higher in the sEMD group than in the bEMD group (6.4 mg/L vs. 4.5 mg/L, *p* = 0.007). Patients with sEMD also exhibited higher frequencies of thrombocytopenia, elevated lactate dehydrogenase (LDH), and renal insufficiency, consistent with numerous domestic and international studies [20,21], and in this study, multivariate analysis further demonstrated that each 1 mg/L increase in β2-MG was associated with a 7.9% and 8.0% higher risk of progression or death (PFS) and overall mortality (OS), respectively. This aligns with the designation of β2-MG as a continuous adverse prognostic variable in the International Myeloma Working Group (IMWG) risk stratification model [7]. He et al. [22] previously reported that newly diagnosed MM patients with EMD commonly presented with high tumor burden, severe anemia, thrombocytopenia, and renal impairment. Although their analysis did not distinguish between bEMD and sEMD, our results indicate that these high-risk features are more pronounced in the sEMD subset. Moreover, the proportion of CD56-negative plasma cells was higher in the sEMD group than in the bEMD group (72.2% vs. 51.9%), a difference that approached statistical significance (*p* = 0.053). CD56 is a key adhesion molecule mediating the interaction between myeloma cells and bone marrow stromal cells. Loss of CD56 expression has been shown to reduce tumor cell retention within the marrow niche and facilitate extramedullary dissemination, providing a potential biological basis for the heightened extramedullary propensity observed in sEMD [23].

Cytogenetic abnormalities are strongly associated with the development of EMD in MM [24]; however, whether the prognostic disparity between bEMD and sEMD subtypes is attributable to distinct cytogenetic profiles remains not fully defined. High-risk cytogenetic abnormalities, including t(4;14), del(17p), and 1q21 gain or amplification, have been reported in patients with extramedullary manifestations of MM, although their specific contributions to extramedullary dissemination remain incompletely defined [25]. In a large cohort reported by Xu et al. [26] RB1 deletion (32.3%, 444/1376) and 1q21 gain/amplification (29.6%, 498/1684) were the most frequent cytogenetic aberrations among MM patients with EMD. Notably, 1q21 gain/amplification was the predominant abnormality in the sEMD subgroup, observed in 32.2% (141/438) of cases. In the present study, the incidence of 1q21 gain/amplification was 49.0% in the bEMD group vs. 30.0% in the sEMD group (*p* = 0.097). Although this difference did not reach statistical significance, the observed trend is consistent with the findings of Xu and colleagues [26]. Their data further corroborate that both TP53 deletion and RB1 deletion occur at significantly higher frequencies in EMD patients compared with those without extramedullary involvement. Within our cohort, the prevalence of TP53 deletion (13.3%) and RB1 deletion (31.0%) was notably higher in the sEMD group than in the bEMD group, reinforcing the notion that adverse cytogenetic features contribute to the aggressive phenotype of sEMD. TP53 deletion, now codified as a core component of the “double-hit” or “triple-hit” high-risk definition by the International Myeloma Working Group (IMWG) [27], exhibited clear prognostic relevance in our analysis. Among 79 patients with complete cytogenetic data, those harboring TP53 deletion exhibited markedly shorter median PFS (8.0 vs. 21.0 months) and OS (13.0 vs. 39.0 months). In multivariate analysis, TP53 deletion emerged as an independent predictor of both PFS (HR = 2.88, *p* = 0.016) and OS (HR = 3.42, *p* = 0.014). These findings align with those of Flynt et al. [28], who reported that TP53 alterations drive uncontrolled proliferation, chemoresistance, and enhanced invasiveness, establishing them as powerful determinants of poor clinical outcome. Although the incidence of TP53 deletion did not differ significantly between the sEMD and bEMD groups in our study, it is possible—as suggested by Matsubara et al. [5]—that TP53 loss may synergize with additional molecular aberrations (e.g., loss of CD56) to exacerbate disease progression in the sEMD subtype. This hypothesis warrants validation in larger, prospective cohorts.

Patients in the bEMD group exhibited significantly longer median PFS and OS than those in the sEMD group, consistent with previous reports [4,29]. In our cohort, patients with multisite extramedullary involvement had a median PFS of 17.0 months and median OS of 27.0 months, approximating the 18.0 and 36.0 months, respectively, reported by Zhao et al. [21]. Notably, thrombocytopenia (PLT < 100 × 10^9^/L) emerged as an independent adverse prognostic factor for both PFS and OS in MM patients with EMD. Although data specifically addressing the prognostic impact of thrombocytopenia in EMD remain relatively limited, a retrospective analysis of 241 patients with newly diagnosed MM (NDMM) similarly demonstrated that thrombocytopenia was independently associated with shorter OS and PFS [30]. The underlying mechanisms are not fully understood; however, Swieringa et al. [31] reported acquired platelet secretion defects in MM, wherein excess serine within the tumor microenvironment impairs megakaryocyte maturation and reduces platelet granule release, thereby compromising hemostatic function.

Using routinely available clinical parameters, we developed a simplified prognostic risk scoring system incorporating β2-microglobulin, thrombocytopenia, and multisite extramedullary infiltration. This model stratified patients into low-risk (0–2 points) and high-risk (3–4 points) groups, with significantly different PFS (27.0 vs. 10.0 months) and OS (54.0 vs. 22.0 months; *p* < 0.001 and *p* = 0.008, respectively). The variable selection and weight assignment of our model align closely with the multisite EMD prognostic model proposed by Zhao et al. [21], which similarly identified thrombocytopenia and multisite involvement as independent adverse determinants.

It should be emphasized that the objective of the present study was to develop a prognostic tool based exclusively on baseline characteristics obtained at initial diagnosis, intended for use in the pre-treatment setting. Compared to established staging systems such as ISS or R2-ISS [27], which were developed for the general MM population, our scoring system was specifically designed for patients with EMD, a subgroup characterized by substantial clinical heterogeneity and poor outcomes. The selected variables,β2-microglobulin, platelet count, and the number of extramedullary involvement sites, are readily available at diagnosis and do not require specialized molecular testing, supporting its potential applicability in routine clinical practice. Importantly, this model was developed as a pretreatment risk stratification tool rather than a treatment-selection algorithm. In clinical settings, patients identified as high risk at diagnosis may benefit from closer monitoring, early consideration of intensified therapeutic approaches, enrollment into clinical trials, or more frequent response assessment. Conversely, the score may provide clinicians with an objective baseline assessment to complement existing staging systems and facilitate individualized management strategies.

However, several limitations should be acknowledged. First, this was a single-center retrospective study with a relatively small sample size, particularly within the sEMD subgroup, which may limit statistical power and generalizability. Second, treatment-related variables were not incorporated into the prognostic model because of substantial heterogeneity in therapeutic strategies across the 15-year study period. Therefore, the score should not be interpreted as predicting treatment efficacy or guiding selection of a specific regimen. Third, although internal validation demonstrated the stability of the model, external validation in larger multicenter cohorts with standardized imaging, molecular profiling, and treatment information remains necessary. Future studies integrating cytogenetic, molecular, and immune microenvironmental features may further improve risk prediction in patients with EMD.

## 5. Conclusions

This study identifies sEMD as a clinically aggressive MM subtype with inferior outcomes compared with bEMD. Thrombocytopenia, elevated β2-microglobulin, multisite extramedullary involvement, and TP53 deletion were associated with adverse prognosis. A simple risk score incorporating three readily available clinical parameters enabled pretreatment risk stratification of patients with EMD. External validation and integration of molecular and immune features may further refine prognostic assessment.

## Figures and Tables

**Table 1:** Comparison of clinical characteristics between bEMD and sEMD groups among 118 patients with multiple myeloma complicated with extramedullary disease.

Clinical Features, *n* (%)	bEMD (*n* = 72)	sEMD (*n* = 46)	χ^2^/z	*p*
Age [years, M (range)]	59.5 (52–66)	61.0 (55.0–69.3)	−1.403	0.161
Gender [*n* (%)]			6.932	0.008
male	46 (63.9)	18 (39.1)		
female	26 (36.1)	28 (60.9)		
β2-MG [mg/L, M (range)]	4.5 (2.8–7.4)	6.4 (4.0–10.0)	−2.679	0.007
Ki-67 proliferation index of tumor cells [%, M (range)]	40.0 (15.00–62.50)	50.0 (17.50–70.00)	−0.339	0.734
Thrombocytopenia [*n* (%)]	8 (11.1)	11 (23.9)	3.405	0.065
Anemia [*n* (%)]	35 (48.6)	29 (63.0)	2.355	0.125
Elevated LDH [*n* (%)]	14 (19.4)	16 (34.8)	3.483	0.062
Renal insufficiency [*n* (%)]	15 (20.8)	16 (34.8)	2.820	0.093
Multifocal extramedullary infiltration [*n* (%)]	18 (25.0)	18 (39.1)	2.643	0.104
BMPC ≥ 50% [*n*/N (%)]	52.2 (35/67)	64.3 (27/42)	1.528	0.216
NDMM with EMD [*n* (%)]	61 (84.7)	27 (58.7)	10.028	0.002
RRMM with EMD [*n* (%)]	11 (15.3)	19 (41.3)	10.028	0.002
M protein type [*n* (%)]			1.429	0.489
IgA	15 (20.8)	12 (26.1)		
IgG	33 (45.8)	16 (34.8)		
Others	24 (33.3)	18 (39.1)		
Light chain type [*n* (%)]			-	0.103
κ	29 (40.3)	10 (21.7)		
λ	38 (52.8)	32 (69.6)		
Non-secretory type	5 (6.9)	4 (8.7)		
DS stage [*n* (%)]			0.177	0.674
I + II	9 (12.5)	7 (15.2)		
III	63 (87.5)	39 (84.8)		
ISS stage [*n* (%)]			2.244	0.326
I	18 (25.0)	9 (19.6)		
II	25 (34.7)	12 (26.1)		
III	29 (40.3)	25 (54.3)		
IGH-R [*n* (%)]	60.8 (31/51)	58.6 (17/29)	0.036	0.849
Del TP53 [*n* (%)]	12.2 (6/49)	13.3 (4/30)		1.000
Del RB1 [*n* (%)]	23.9 (11/46)	31.0 (9/29)	0.461	0.497
Gain/Amp 1q21 [*n* (%)]	49.0 (24/49)	30.0 (9/30)	2.756	0.097
CD56^−^ [*n* (%)]	51.9 (28/54)	72.2 (26/36)	3.735	0.053
Induction [*n*(%)]				1.000
PIs	21/71 (29.6)	13/44 (29.5)		
IMiDs	3/71 (4.2)	1/44 (2.3)		
PIs + IMiDs	36/71 (50.7)	23/44 (52.3)		
CD38 mAb	11/71 (15.5)	7/44 (15.9)		
ASCT [*n* (%)]	12 (16.7)	4 (8.7)	1.522	0.217

Abbreviations: IGH-R: IGH rearrangement; Del TP53: TP53 deletion; Del RB1: RB1 deletion; Gain/Amp 1q21: 1q21 gains (3 copies) or amplifications (4 copies); CD56^−^: CD56 negative; ASCT: autologous stem cell transplantation.

**Table 2:** Univariate and multivariate Cox analyses for PFS in EMD patients (*n* = 118).

Risk	*n*	Univariate		Multivariate	
		HR (95% CI)	*p*	HR (95% CI)	*p*
β2-MG (mg/L)	118	1.07 (1.03–1.10)	<0.001	1.08 (1.05–1.11)	<0.001
Thrombocytopenia	19/118	2.91 (1.68–5.03)	<0.001	2.57 (1.37–4.82)	0.003
Multifocal extramedullary infiltration	36/118	2.51 (1.58–3.98)	<0.001	2.23 (1.34–3.71)	0.002

**Table 3:** Univariate and multivariate Cox analyses for OS in EMD patients (*n* = 118).

Risk	*n*	Univariate		Multivariate	
		HR (95% CI)	*p*	HR (95% CI)	*p*
β2-MG (mg/L)	118	1.08 (1.05–1.11)	<0.001	1.08 (1.05–1.12)	<0.001
Thrombocytopenia	19/118	2.60 (1.42–4.76)	0.001	2.68 (1.45–4.94)	0.002

**Table 4:** Prognostic risk scoring system.

Risk	Definition	Score
β2-MG(mg/L)	<3.5	0
	3.5–5.5	1
	>5.5	2
Thrombocytopenia	<100 × 10^9^/L	1
Multifocal extramedullary infiltration	≥2	1

**Table 5:** Bootstrap validation of variables in the prognostic scoring system (PFS model).

Risk	B	PFS HR (95% CI)	*p*
Multifocal extramedullary infiltration	0.833	2.30 (1.43–3.71)	<0.001
Thrombocytopenia	0.852	2.35 (1.33–4.13)	0.003
β2-MG score	0.126	1.13 (0.87–1.48)	0.352

**Table 6:** Bootstrap validation of variables in the prognostic scoring system (OS model).

Risk	B	OS HR (95% CI)	*p*
Multifocal extramedullary infiltration	0.289	1.33 (0.69–2.51)	0.394
Thrombocytopenia	0.950	2.59 (1.02–8.99)	0.044
β2-MG score	0.177	1.19 (0.86–1.66)	0.286

## Data Availability

Due to institutional privacy policy requirements, deidentified individual participant data that underlie the results reported in this article, including text, tables, figures, and appendices, will be made available upon reasonable request to the corresponding author (qinxiaoqi6@163.com), beginning 3 months and ending 5 years after publication.

## Supplementary Materials

The following supporting information can be downloaded at: https://www.mdpi.com/article/10.3390/cancers18152484/s1. Figure S1: Survival outcomes according to combined classification of disease status and extramedullary disease subtype; Table S1: Univariate Cox analysis of additional factors for PFS in EMD patients (n = 118); Table S2: Univariate Cox analysis of additional factors for OS in EMD patients (n = 118); Table S3: Assessment of proportional hazards assumption for the PFS Cox regression model; Table S4: Comparison of discrimination and predictive performance between the prognostic risk scoring system and ISS staging.
