# Supplementary material for: Clinical Characteristics and Prognostic Analysis of Extramedullary Disease in Multiple Myeloma: A 15-Year Retrospective Cohort Study
Source: Cancers (Basel). 2026 Aug 3;18(15):2484. doi: 10.3390/cancers18152484 (PMC13465512; doi:10.3390/cancers18152484)
Supplement: Supplementary file 1 [file cancers-18-02484-s001.zip › cancers-4419443-supplementary.pdf]

Supplementary data For

Clinical Characteristics and Prognostic Analysis of Extramedullary Disease in Multiple Myeloma: A 15-Year Retrospective Cohort Study

FigureS1
Survival outcomes according to combined classification of disease status and extramedullary disease subtype

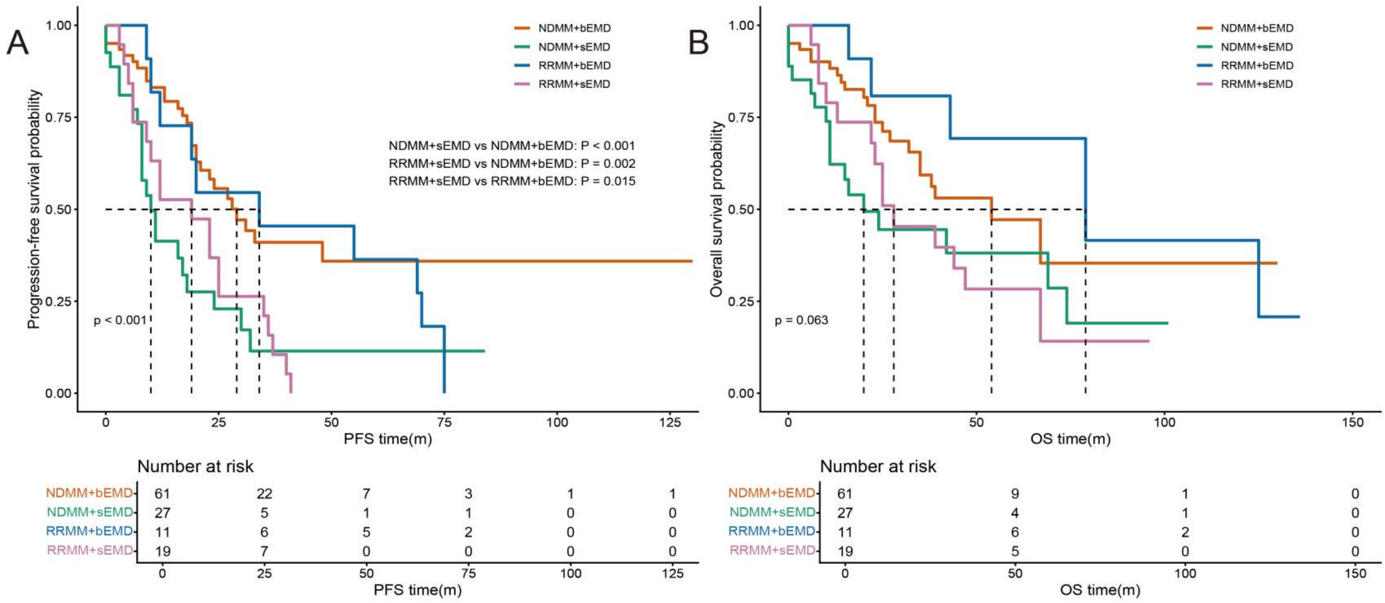

Table S1 Univariate Cox analysis of additional factors for PFS in EMD patients (n = 118)

Risk	events	n	Univariate	
			HR (95% CI)	*P*
Age>60	80	57/118	1.89(1.21–2.94)	0.005
Age>65	80	32/118	1.68(1.04–2.72)	0.035
EMD (sEMD)	80	46/118	2.43 (1.56–3.79)	<0.001
BMPC infiltration	72	62/109	1.84 (1.13–3.00)	0.014
Anemia	80	64/118	1.70 (1.08–2.68)	0.021
Elevated LDH	80	30/118	1.66 (1.04–2.67)	0.035
IGH-R	52	48/80	0.79 (0.45–1.37)	0.402

Del TP53	55	10/79	3.31 (1.50–7.33)	0.003
Del RB1	53	20/75	1.04 (0.54–1.98)	0.915
Gain/Amp 1q21	53	33/79	1.44 (0.83–2.51)	0.196
induction regimes	78	18/115	1.28 (0.65–2.52)	0.468
Transplantation	9	16/118	0.56 (0.28–1.13)	0.107

Table S2 Univariate Cox analysis of additional factors for OS in EMD patients (n = 118)

Risk	events	n	Univariate	
			HR (95% CI)	*P*
Multifocal extramedullary infiltration	60	36/118	1.48 (0.87 – 2.52)	0.144
Age>60	60	57/118	2.92(1.70 – 5.01)	<0.001
Age>65	60	32/118	2.54(1.47 – 4.40)	<0.001
EMD (sEMD)	60	46/118	1.94 (1.16–3.23)	0.011
BMPC infiltration	53	62/109	1.51 (0.86–2.64)	0.152
Anemia	60	64/118	1.66 (0.99–2.80)	0.057
Elevated LDH	60	30/118	1.05 (0.59–1.87)	0.856
IGH-R	37	48/80	0.91 (0.47–1.79)	0.793
Del TP53	39	10/79	3.43 (1.35–8.72)	0.010
Del RB1	37	20/75	0.90 (0.39–2.05)	0.799
Gain/Amp 1q21	38	33/79	1.74 (0.91–3.34)	0.096
induction regimes	57	18/115	1.36 (0.60–3.07)	0.456
Transplantation	2	16/118	0.14 (0.03 – 0.59)	0.007

Table S3. Assessment of proportional hazards assumption for the PFS Cox regression model

Variable	χ^2	df	P value
β2-MG (mg/L)	0.232	1	0.630
Thrombocytopenia	4.656	1	0.031

Multifocal extramedullary infiltration	0.788	1	0.375
Global test	5.339	3	0.149

Table S4.Comparison of discrimination and predictive performance between the prognostic risk scoring system and ISS staging

Outcome	Model	C-index(SE)	AIC	LRT χ^2	P value	3-year AUC	5-year AUC
PFS	Prognostic risk scoring system	0.602 (0.026)	625.08	14.192	<0.001	0.622	0.640
	ISS stage	0.531 (0.032)	641.27	—	—	0.583	0.528
OS	Prognostic risk scoring system	0.588 (0.034)	480.31	0.211	0.646	0.588	0.526
	ISS stage	0.601 (0.035)	482.10	—	—	0.603	0.571
